# Plasma Levels of Metalloproteinase 3 (MMP-3) and Metalloproteinase 7 (MMP-7) as New Candidates for Tumor Biomarkers in Diagnostic of Breast Cancer Patients

**DOI:** 10.3390/jcm12072618

**Published:** 2023-03-30

**Authors:** Paweł Ławicki, Paweł Malinowski, Joanna Motyka, Michał Ławicki, Aleksandra Kicman, Monika Kulesza, Ewa Gacuta, Tomasz Guszczyn, Marcin Januszkiewicz, Monika Zbucka-Krętowska, Sławomir Ławicki

**Affiliations:** 1 Department of Population Medicine and Lifestyle Diseases Prevention, Medical University of Bialystok, 15-269 Bialystok, Poland; 2 Department of Oncological Surgery, Bialystok Oncology Center, 15-276 Bialystok, Poland; 3 Department of Aesthetic Medicine, Medical University of Bialystok, 15-267 Bialystok, Poland; 4 Department of Perinatology, University Clinical Hospital of Bialystok, 15-276 Bialystok, Poland; 5 Department of Pediatric Orthopaedics and Traumatology, Medical University of Bialystok, 15-089 Bialystok, Poland; 6 Department of Gynecological Endocrinology and Adolescent Gynecology, Medical University of Bialystok, 15-276 Bialystok, Poland

**Keywords:** breast cancer, luminal a subtype, luminal b her2-negative subtype, *fibroadenoma*, mmp-3, mmp-7, ca 15-3

## Abstract

Matrix metalloproteinases (MMPs) are a group of enzymes that mediate both physiological and pathological processes such as carcinogenesis. The role of matrix metalloproteinase-3 (MMP-3) and (MMP-7) in the pathogenesis of breast cancer (BC) has been demonstrated, suggesting that they may be considered as potential markers of this condition. The aim of this study was to assess plasma concentrations and diagnostic utility of MMP-3 and MMP-7 in 100 patients with early-stage breast cancer with Luminal A subtype or Luminal B HER-negative subtype, before and after surgical treatment, and in the following control groups: patients with a benign tumor (*fibroadenoma*) and healthy subjects. The concentrations of MMP-3 and MMP-7 were referenced to the levels of the widely recognized marker for BC diagnosis CA 15-3. MMP-3 and MMP-7 was measured by ELISA method and CA 15-3 by CMIA. Plasma levels of MMP-7 were significantly higher in Luminal A and Luminal B HER2-negative subtype breast cancer patients as compared to the healthy group. MMP-7 demonstrated comparable but mostly higher to CA 15-3 or MMP-3 values of diagnostic sensitivity, specificity, positive and negative predictive values and AUC (0.6888 for Luminal A subtype; 0.7612 for Luminal B HER2-negative; 0.7250 for BC total group, respectively) in the groups tested. The combined use of the tested parameters resulted in a further increase in diagnostic criteria and AUC. These results suggest the usefulness of combining MMP-7 with CA 15-3 in the diagnostics of breast cancer, especially in Luminal B HER2-negative subtypes patients, as a new candidate for tumor markers.

## 1. Introduction

Breast cancer is the most frequent type of female neoplasm and the first (in low-income countries) or second (in high-income countries) most frequent cause of death. Currently, there is a steady increase in the incidence of breast cancer [1]. By 2040, the incidence is projected to reach about 3.19 million new cases [1]. The groups at highest risk for breast cancer are perimenopausal and postmenopausal women—between the ages of 45 and 70. An increasing trend in incidence was also recently observed among younger women of reproductive age, of 20 to 40 years [2]. Many studies have noted an increase in the 5-year survival rate of patients in many countries, but the percentage still varies from 90% to 20%, mainly depending on the stage of the cancer at the time of detection [2,3,4,5]. Increased survival rates are associated with the introduction of targeted treatment, improved diagnostic methods, including the introduction of screening (mammography) in a group of women at increased risk of breast cancer allowing earlier detection of changes, and education of the public [2,5]. However, what has remained unchanged over the years is the correlation between the higher survival rates of patients with positive estrogen and progesterone receptor status than patients who have not shown expression of those receptors. These differences are believed to be driven by the less aggressive nature of tumors with positive hormone receptor expression, as well as the availability of adjuvant hormonal therapies, which are only available for hormone-dependent types of breast cancer [5].

Metalloproteinases (MMPs) are involved in many physiological and pathological processes, mediating in cell proliferation, migration, invasion, differentiation, apoptosis, inflammation and angiogenesis—by degradation of many extracellular matrix (ECM) proteins [6]. In carcinogenesis, the degradation of ECM components through the proteolytic activity of MMPs can enhance the growth of primary tumor and angiogenesis and can promote metastases formation in distant loci [7,8,9,10]. Numerous studies have confirmed the significant involvement of MMPs in pathogenesis of breast cancer.

Overexpression of MMP3 and its role as a prognostic factor have been reported in several cancers, including breast cancer [11,12]. In vitro and in vivo study has demonstrated the involvement of MMP-3 in the metastasis of breast cancer cell line 4T1 [13]. A correlation between tissue expression levels of MMP-3 and the degree of malignancy of the lesion has also been reported, suggesting its potential as a biomarker capable of differentiating early malignancy from dysplasia [14]. Additionally, there is an article that investigates the potential of circulating MMP3 as a prognostic marker for breast cancer, where elevated serum levels of MMP3 were reported in breast cancer patients. The levels also correlated with the stage and malignancy of the tumor [15]. In addition, the authors suggested the utility of MMP-3 as a marker for detecting recurrence, as elevated levels of MMP3 were observed 3 months after primary surgery [15].

Several available papers on MMP-7 have demonstrated its higher expression in breast cancer with respect to healthy controls [16], as well as an association of higher expression with faster tumor growth rates, increased tumor cell invasiveness, metastasis to distant locations, shorter relapse-free interval and shorter 5-year survival [17,18,19,20]. Work by Davidson et al. [21] and Piskór et al. [22] also suggested the utility of determining MMP-7 levels or its expression in serum and cellular effusion in breast cancer as a diagnostic marker and as a differentiation marker from benign lesions [21,22]. The diagnostic potential of MMP-7 has also been noted for several gynecologic cancers such as ovarian cancer [21,23,24,25], endometrial cancer [21,26] and cervical cancer [11,27,28,29].

An increasing number of works are starting to show that MMPs can serve as diagnostic and prognostic biomarkers in early detection and risk assessment, as well as markers of therapy efficacy in breast cancer treatment [30,31,32,33,34]. Given the suggested role of MMP-3 and MMP-7 in the pathogenesis of breast cancer and their diagnostic potential as demonstrated in research papers, we decided to test them as early tumor markers of breast cancer.

In this paper, we present a continuation of the evaluation of MMPs as new tumor markers for breast cancer, this time by assessing two metalloproteinases MMP-3 and MMP-7 as a diagnostic marker of early-stages breast cancer with Luminal A or Luminal B HER-negative subtypes. The aims of the study were to determine the plasma levels of selected metalloproteinases in comparison with the tumor marker CA15-3 in breast cancer patients and with reference to two control groups: patients with benign lesions (*fibroadenoma*) and healthy women and to assess their diagnostic utility as individual markers and as a combined parameter with CA 15-3. According to the results obtained by our team on MMP-3 and MMP-7, they may appear to be useful as new biomarkers in the primary diagnosis of patients with early stage breast cancer (TNM stage I) and the most common subtypes—Luminal A and Luminal B HER2-negative.

## 2. Materials and Methods

A group of 100 female patients with breast cancer (BC) diagnosed and treated at the Oncology Center in Bialystok was used as a study group. The criteria for including patients in the study covered available medical history data (age, race—Caucasian, menopausal status, breast cancer risk factors, family history) and clinicopathological results (stage of cancer in TNM classification, histological type, receptor status involving estrogen (ER), progesterone (PR) receptors and Ki67 proliferative index). Histopathological evaluation for each patient was assessed by preoperative breast biopsy specimens or intraoperatively obtained tumor tissue samples. For the purposes of the study, patients were separated into two groups based on their receptor status—a group of patients with only the Luminal A type of breast cancer and a group of patients with only the Luminal B type of breast cancer without the presence of the HER2 receptor (Luminal B HER2-negative). The diagnosis of stage I breast cancer by the TNM classification was established in all participating patients. We included two control groups in the study: a group of 50 patients with benign *fibroadenoma* breast lesions and a group of 50 healthy women. The control groups were matched in terms of the age of the participants to the age of the patients included in the study group. Table 1 outlines the specific characteristics of all studied groups.

The first qualification of patients in the groups with breast cancer and benign lesion was determined via gynecological examination. The next stage of qualification involved confirmatory examinations conducted by an oncology specialist with imaging methods (ultrasound/MRI depending on the individual case) and complementary laboratory examinations. The next stage of verification involved screening of qualified patients by the oncologist-surgeon or oncologist in charge of the patient’s treatment by conducting additional tests to assess the patient’s condition—blood tests, ultrasound, mammography, and in some cases also other imaging tests, such as magnetic resonance imaging. Selection of the study and control group of patients with benign lesions, preoperative therapeutic treatments were given by the hospital facility following the latest clinical practice directives for breast cancer treatment. Out of the study group were eliminated patients suffering from malignant lesions who had received pre-operative adjuvant therapy. For the post-operative evaluation of the studied parameters, the blood specimens were collected from the patients over a period of 4–6 weeks after the surgical treatment and prior to the introduction of further targeted treatment.

An active inflammatory process was ruled out in all participants of the study (women with cancer, benign lesion and healthy women) by appropriately selected laboratory examinations, among them a full blood count, evaluation of the blood smear, determination of C-reactive protein levels and other chosen enzymatic tests. Women classified as healthy control were recruited by their family physician and addressed to a gynecologist, who reconfirmed their applicability to participate in the investigation on a routine check-up appointment at the Department of Gynecology of the University Clinical Hospital in Bialystok. For each contestant, the menopausal status was defined—all of the women were postmenopausal.

Plasma extracted from venous blood collected for the anticoagulant—lithium heparin—was used as the study material. Blood samples were obtained from the subjects and within 30 min post collection centrifuged with 1810× *g* force for 10 min. The separated plasma was further preserved at −85 °C up to the assay day.

Plasma MMP-3 and MMP-7 concentrations were measured with the use of enzyme-linked immunosorbent assay (ELISA) (Human Total MMP-7 Quantikine ELISA Kit Cat. No. DMP700, Human Total MMP-3 Quantikine ELISA Kit Cat. No. DMP300, R&D Systems Inc., Minneapolis, MN, USA). Arrangements of the assay were carried out following the manufacturer’s instructions enclosed with the ELISA kits with duplicate sample determinations for the reference curve and test specimens. Intra- and inter-laboratory precision were predetermined by the vendor (MMP-7: 3.7%, 4.1%; MMP-3: 6.1%, 7.0%, respectively). For measurement of CA 15-3 plasma levels, we used a chemiluminescent microparticle immunoassay (CMIA) (Abbott, Chicago, IL, USA) according to the manufacturer’s protocols.

### Statistical Analysis

Statistical analysis of data was performed using PQStat ver.1.8.2 PQStat Software, (Poznan, Poland) and GraphPad Prism 9.3.1. for Windows, GraphPad Software (San Diego, CA, USA).

The Shapiro–Wilk test showed the non-normality of the data distribution; hence, the statistical analysis was based on non-parametric tests—the comparison of independent groups was performed by the Kruskal–Wallis’s test with the Conover–Iman post hoc test (when more than 2 groups were compared) or the U Mann–Whitney test (for comparison of 2 groups); the correlation between the studied parameters was assessed by r Spearman test. Evaluation of changes between pre- and post-operative concentrations was performed with the Wilcoxon matched pairs test. The evaluation of diagnostic criteria (sensitivity, specificity, positive and negative predictive values, diagnostic power of the test) was based on receiver-operating characteristic curve (ROC) analysis for tested parameters investigated as single parameter as well as combinations of parameters. Optimal cut-off values were defined by a point on the ROC curve at the minimum distance from the upper-left corner of the graph and reached 4.34 ng/mL for MMP-3, 1.274 ng/mL for MMP7 and 19.74 IU/mL for CA 15-3.

## 3. Results

### 3.1. Plasma Concentration of Tested Parameters

**Figure 1:**
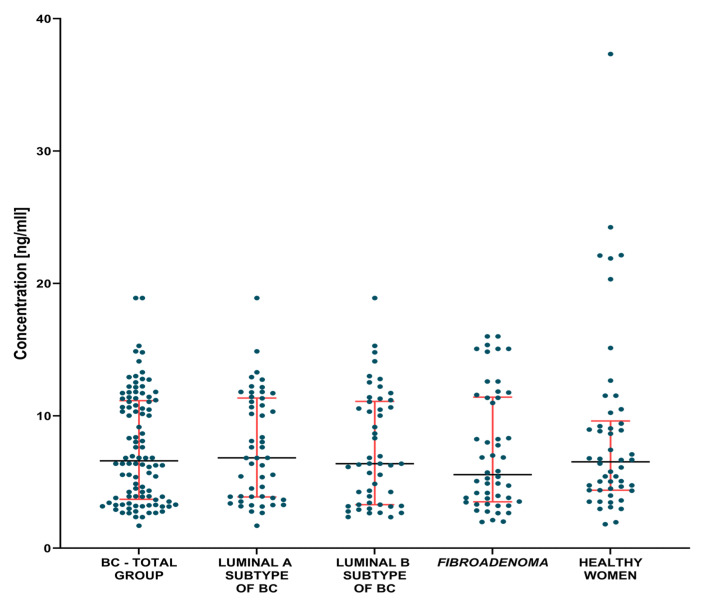
MMP-3 plasma concentrations (with marked median and interquartile range) in all tested groups: patients with BC, *fibroadenoma* subjects and healthy women.

**Figure 2:**
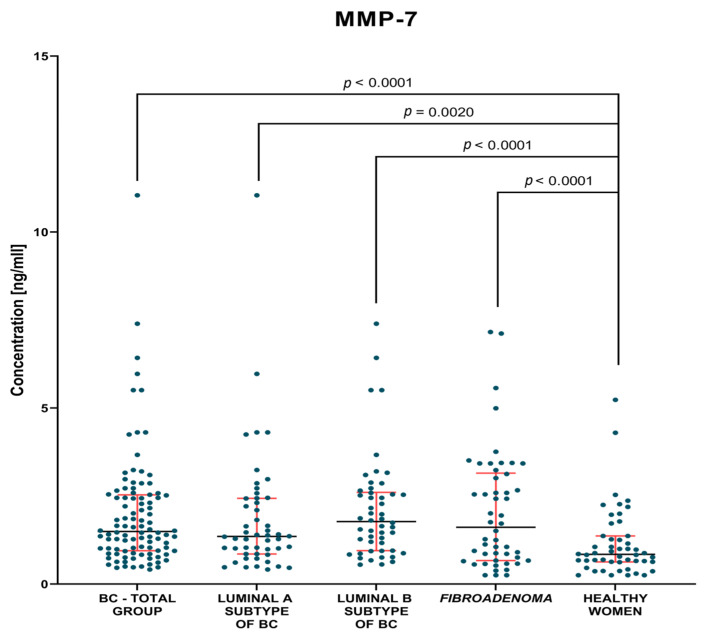
MMP-7 plasma concentrations (with marked median and interquartile range) in all tested groups: patients with BC, *fibroadenoma* subjects and healthy women. Significant statistical differences have been marked on the figure.

**Figure 3:**
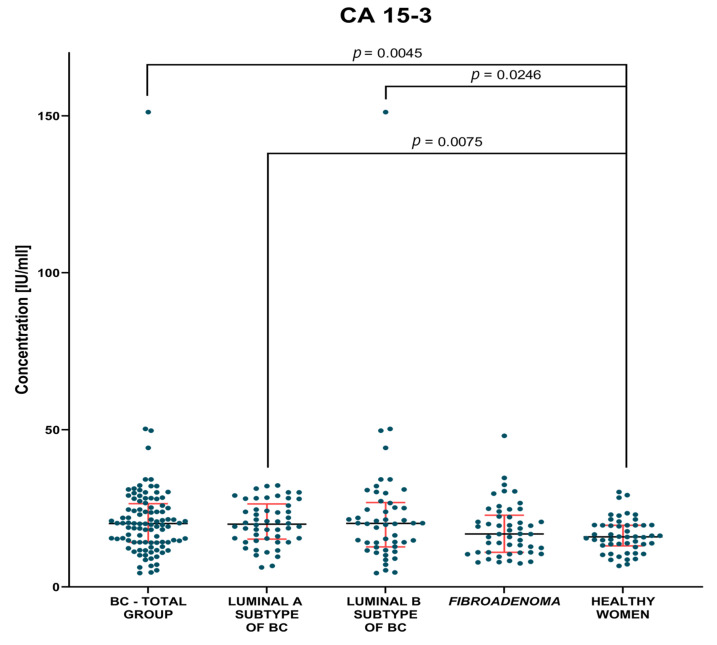
CA 15-3plasma concentrations (with marked median and interquartile range) in all tested groups: patients with BC, *fibroadenoma* subjects and healthy women. Significant statistical differences have been marked on the figure.

**Figure 4:**
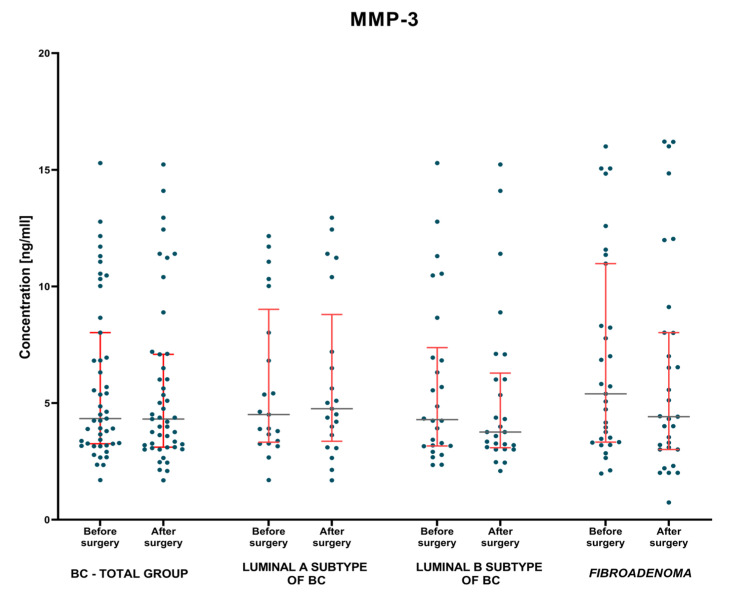
MMP-3 plasma concentrations (with marked median and interquartile range) in patients (total group and subgroups) with BC and *fibroadenoma* subjects before and after surgery.

**Figure 5:**
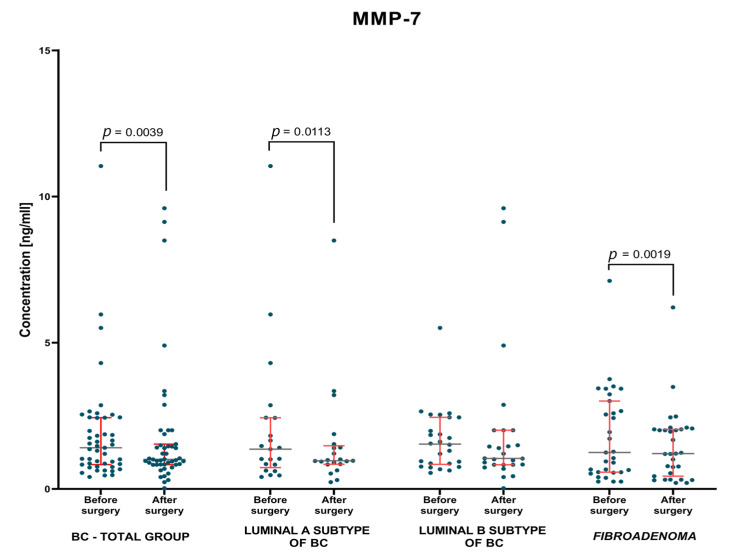
MMP-7 plasma concentrations (with marked median and interquartile range) in patients (total group and subgroups) with BC and *fibroadenoma* subjects before and after surgery. Significant statistical differences have been marked on the figure.

**Figure 6:**
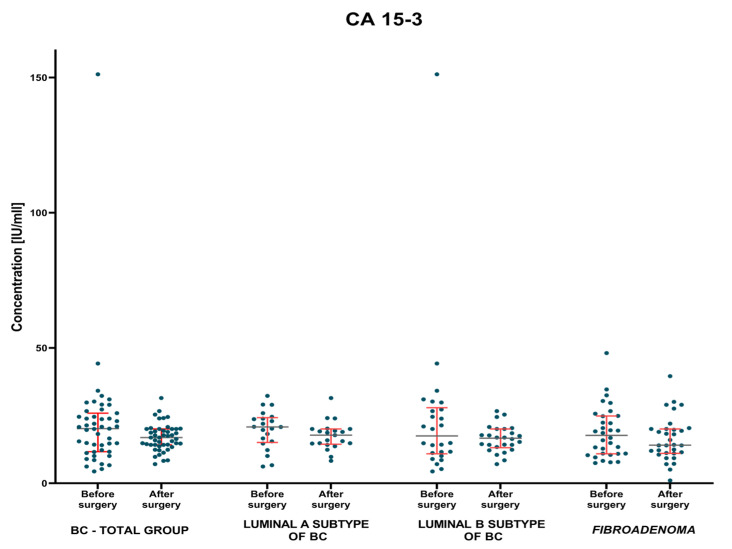
CA 15-3 plasma concentrations (with marked median and interquartile range) in patients (total group and subgroups) with BC and *fibroadenoma* subjects before and after surgery.

The evaluation of the plasma levels of MMP-3, MMP-7 and the CA 15-3 marker in breast cancer patients compared to patients with benign lesions and compared to a control group of healthy women before and after surgery are shown in Table 2 and Table 3 and Figure 1, Figure 2, Figure 3, Figure 4, Figure 5 and Figure 6.

#### 3.1.1. Preoperative Groups of Patients

Analysis of data showed significantly higher concentrations of MMP-7 (median 1.49 ng/mL) and CA 15-3 (median 20.16 IU/mL) in the BC-total group of patients than in the control group of healthy women (0.84 ng/mL, *p* < 0.0001; 15.95 IU/mL, *p* = 0.0045, respectively). Additionally, significantly higher concentrations of MMP-7 (1.62 ng/mL) were observed in *fibroadenoma* group in comparison to healthy women group (*p* < 0.0001).

In the groups of Luminal A and Luminal B HER2-negative BC patients, we found significantly higher concentrations of MMP-7 (1.35 ng/mL, *p*. = 0.002; 1.78 ng/mL, *p* < 0.001, respectively) and the comparative marker CA 15-3 (19.98 IU/mL, *p* = 0.0075; 20.2 IU/mL *p* = 0.0248, respectively) compared to the group of healthy women (Figure 2 and Figure 3). Statistical analysis showed no significant differences between MMP-3 concentrations between the tested Luminal subgroups versus the control groups (Figure 1).

#### 3.1.2. Postoperative Group of Patients—Analysis of Matched Pre- to Postoperative Pairs

The evaluation of changes in the concentrations of the studied parameters and the control marker was carried out on the basis of the non-parametric test for two dependent groups when studying either group of patients with breast cancer and with *fibroadenoma* lesions. The test results are shown in Table 3 and Figure 4, Figure 5 and Figure 6.

It was shown that MMP-7 concentrations, identically to the comparative marker CA 15-3, were significantly decreased in the BC-total patient group, in comparison to the period before and after surgery (*p* = 0.004; *p* = 0.031; respectively). An identical correlation, but only for MMP-7 concentrations, was observed when evaluating concentrations in patients with *fibroadenoma* lesions before and after surgery (*p* = 0.002; Figure 5 and Figure 6). Moreover, it was found that when evaluating each subgroup of BC only concentration of MMP-7 had significantly decreased in patients with Luminal A BC when comparing the preoperative and the postoperative period (*p* = 0.011; Figure 5).

When examining the postsurgical concentration of the tested parameters, the statistical analysis showed that after surgical treatment BC-total patients’ group, Luminal B subgroup and fibroadenoma group of patients had significantly lower concentrations of MMP-3 (4.32 ng/mL, *p* = 0.006; 3.76 ng/mL, *p* = 0.005; 4.42 ng/mL, *p* = 0.037, respectively) (Table 3) compared to healthy women group (6.53 ng/mL).

### 3.2. Evaluation of Correlation by Spearman’s Method

**Figure 7:**
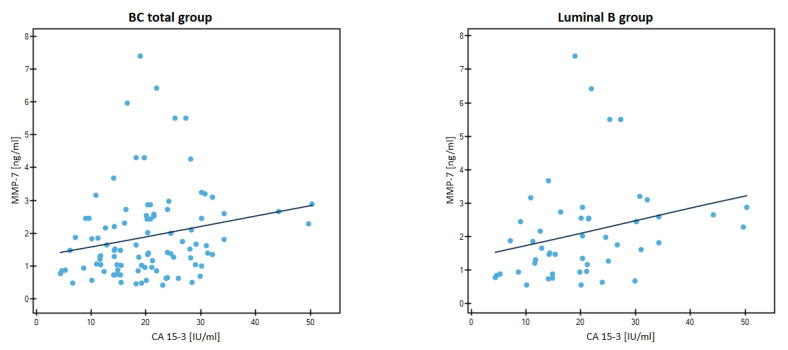
Significant correlations by Spearman’s method between MMP-7 and CA 15-3 in the entire study group and Luminal B HER2-negative subgroup.

**Figure 8:**
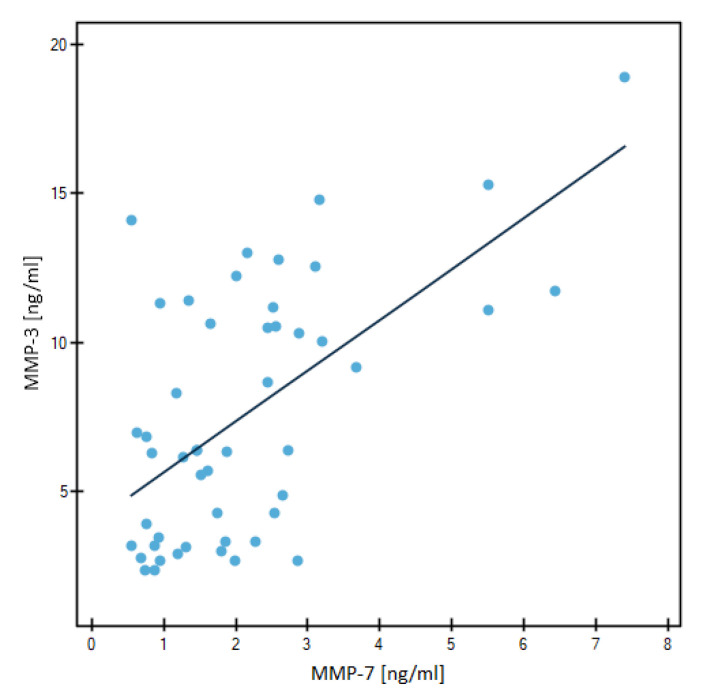
Significant correlations by Spearman’s method between MMP-3 and MMP-7 3 in Luminal B HER2-negative subgroup.

The evaluation of the correlation of the tested parameters and the CA 15-3 marker is presented in Table 4 and in Figure 7 and Figure 8.

The chosen method was Spearman’s non-parametric test. We found significant positive correlations between MMP-7 and CA 15-3 in the BC-total group of patients (r = 0.2583, *p* = 0.0102) and in the Luminal B HER2-negative subgroup of patients (r = 0.3701, *p* = 0.0082). In Luminal B HER2-negative subgroup of patients we also noted a positive correlation between two tested metalloproteinases (r = 0.4901, *p* = 0.0003).

### 3.3. Diagnostic Criteria of MMP-3, MMP-7 and CA 15-3

The following diagnostic criteria—diagnostic sensitivity (SE), diagnostic specificity (SP), positive and negative predictive value (PPV and NPV)—of all tested parameters for total group of BC patients and each subgroup are presented in Table 5.

The highest SE for the BC-total patient group was achieved by MMP-7 (64%), and was higher than the comparative marker CA 15-3 (54%). Furthermore, the combined analysis of MMP-7 and CA 15-3 increased the value of SE to 78%, while the combination of MMP-3, MMP-7 and CA 15-3 increased it to as high as 90%. The highest value of SE in the Luminal A subgroup was also observed for the MMP-7 (58%), and it was higher than the comparative marker (52%). Analysis of combined parameters increased the SE of the tests for MMP-7 + CA 15-3 up to 74% and for MMP-3 + MMP-7 + CA 15-3 up to 94%. The highest value of SE in Luminal B HER2-negative subgroup was observed again for the MMP-7 (70%), and it was higher than the comparative marker (56%). Analysis of combined parameters increased the SE of the tests for MMP-7 + CA 15-3 up to 82% and of MMP-3 + MMP-7 + CA 15-3 up to 86%.

The highest value of SP in the BC-total group was obtained for MMP-3 and CA 15-3, achieving the same value of 80%, although the SP value for MMP-7 remained very close to this value, reaching 74%. Combined analysis of both these parameters did not increase the value of SP.

The highest PPV in the BC-total group was observed for CA 15-3 (84%), while MMP-7 showed a slightly lower value of 83%. The PPV for MMP-3 was also at a comparable level to the previously mentioned parameters, reaching 77%. Combined analysis of tested metalloproteinases in the BC-total group with CA 15-3 slightly increased the value of PPV to 79% for MMP-3 + CA 15-3 combination, and slightly decreased for the combination of MMP-7 + CA 15-3 (80%). The lowest value of PPV was obtained for the three-parametrical combination (68%). The highest values of PPV in both the Luminal A and Luminal B HER2-negative subgroups were observed for the CA 15-3 (72%; 74%, respectively) and MMP-7 (69%; 73%, respectively), which was slightly lower than for the comparative marker. Analysis of parametric panels did not increase the value of PPV of the tests for any of the examined combinations.

The highest NPV for the BC-total patients’ group was achieved by MMP-7 (51%), and it was higher than the comparative marker CA 15-3 (47%). The lowest value of NPV was noted for MMP-3, reaching 37%. The combined parameter analysis of MMP-7 and CA 15-3 increased the value of NPV to 58%, while the three-parametric analysis decreased the NPV value to 41%. The highest value of NPV in the Luminal A subgroup was observed for MMP-7 (64%), and it was slightly higher than the comparative marker (63%). Analysis of combined parameters both for the two-parametric test (MMP-7 + CA 15-3) as well as the three-parametric test (MMP-3 + MMP-7 + CA 15-3) increased NPV to a value of 70%. The highest value of NPV in Luminal B HER-negative subgroup was observed for the MMP-7 (71%) and it was higher than the comparative marker (65%). Analysis of the combined MMP-3 + CA 15-3 parameters increased the NPV of the tests for MMP-7 + CA 15-3 to 77%, while the three-parametric analysis again decreased the value to 50%.

### 3.4. Evaluation of the Diagnostic Power of Tests (ROC Function)

Evaluation of diagnostic power was performed by assessing the area under the ROC curve (AUC). The ROC analysis determines whether a given test makes it possible to distinguish between normal and abnormal results. A diagnostically perfect test completely distinguishes between healthy and sick people (with a sensitivity value of 100% and a specificity value of 100%), in which case the line in the ROC function will completely coincide with the *Y* axis, and the AUC will reach a value 1. A test that cannot distinguish between healthy and sick people, which means that is not diagnostically useful, will have a straight line, inclined at an angle of 45 degrees to the *X*-axis—an AUC value close to 0.5, i.e., the limit of diagnostic usefulness of the test. The detailed parameters of the ROC curve analysis are shown in Table 6.

**Figure 9:**
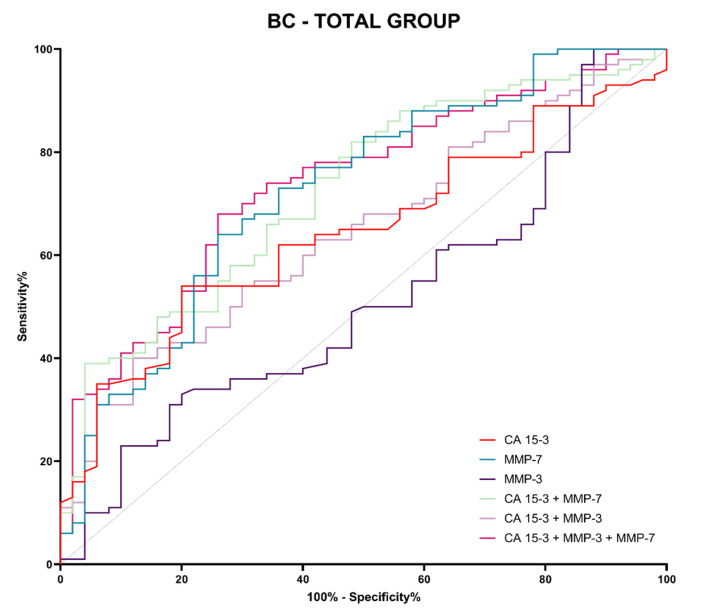
Evaluation of diagnostic power based on area under the ROC curve (AUC) in BC-total group.

In the total BC-total group, the highest AUC value obtained MMP-7 (0.725) which was higher than the comparative marker CA 15-3 (0.6443) (Table 6; Figure 9). Combined analysis of MMP-7 and CA 15-3 did not increase diagnostic power value (0.7246), but for the three-parametric combination there was a slight increment (0.738). In addition, the AUC values of CA 15-3 and all variable analyses with MMP-7 were statistically significantly greater than the limit of diagnostic usefulness of the test (Table 6). Combining MMP-7 with CA 15-3 for both two-component analysis and three-component analysis significantly improved the quality of the assay for the CA 15-3 marker (*p* = 0.0437; *p* = 0.0233, respectively).

**Figure 10:**
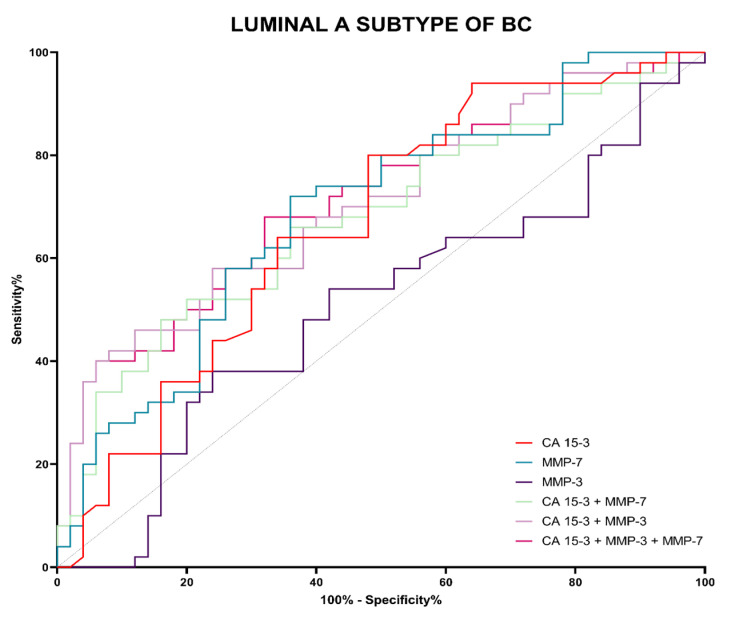
Evaluation of diagnostic power based on area under the ROC curve (AUC) in Luminal A subtype of BC.

The highest value of AUC in the Luminal A subgroup was observed for MMP-7 (0.6888) and was higher than for comparative marker CA 15-3 (0.6672) (Table 5; Figure 10). Analysis of combined parameters shown an increase in diagnostic power of the test, and the highest value was reached in MMP-7 + CA 15-3 (0.7052). However, the test did not show an increase in test quality relative to CA 15-3 (*p* > 0.05). When analyzing this subgroup, MMP-3 and its combinations with other parameters, we did not notice its significance in relation to the limit value of diagnostic usefulness of the test (AUC = 0.5), and hence, these values have been omitted.

**Figure 11:**
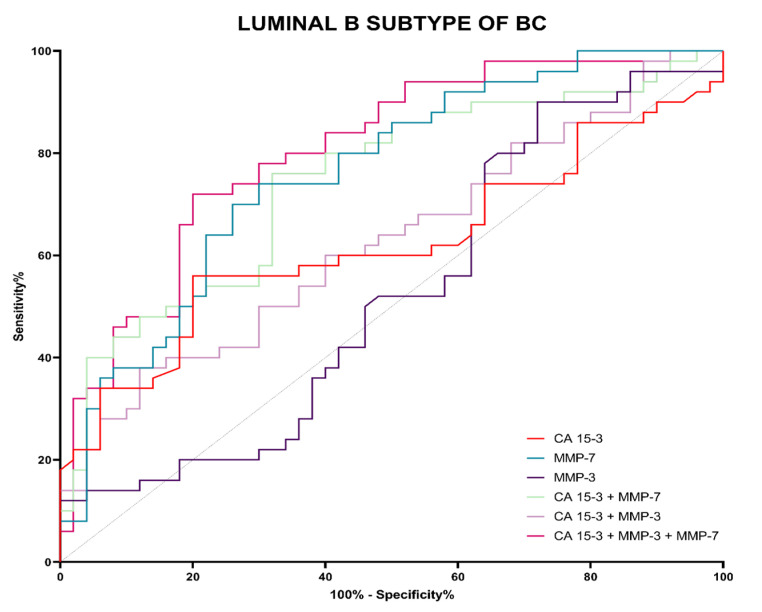
Evaluation of diagnostic power based on the area under the ROC curve (AUC) in Luminal B subtype of BC.

The highest value of AUC in Luminal B HER2-negative subgroup was observed for the MMP-7 (0.7612), and it was higher in value than the comparative marker CA 15-3 (0.6214) (Table 6; Figure 11). Analysis of the combined parameters MMP-7 and CA 15-3 did not increase the diagnostic power of the test (0.7432). Only the three-parametric analysis increased the diagnostic power of the test, reaching a value of 0.8040. Nonetheless, the combination of MMP-7 with CA 15-3, both as a two-parametric and a three-parametric test, significantly improved the quality of the test for CA 15-3 (*p* = 0.0158; *p* = 0.0022, respectively).

## 4. Discussion

Today, breast cancer (BC) is the most common malignancy affecting females globally. Although BC mainly affects perimenopausal and postmenopausal patients, there is an alarming trend of growing incidence in women of reproductive age [2]. Despite the availability of screening and widespread public education about BC, the incidence of the disease continues to increase [1,2,5]. Consequently, new, effective and minimally invasive methods are being sought to diagnose BC faster than before, which will translate into earlier introduction of treatment. Biochemical diagnosis of BC uses tumor markers such as CA 15-3; however, determination of the concentration of this compound is not sufficient to unequivocally diagnose the disease [35]. The incapability of applying CA 15-3 assays for BC diagnosis is due to several important reasons: Firstly, this marker has sensitivity that is too low for detecting BC in situ or in the form of low-stage invasive disease (CA 15-3 concentrations in women with early BC have been shown to coincide with CA 15-3 concentrations in healthy women or those with benign breast lesions). Secondly, the concentrations of this marker in the blood are influenced by several pathological phenomena that can occur in a woman’s body simultaneously with the process of neoplasia, such as lung disease, liver disease, and autoimmune and hematological conditions [35,36]. The search for new potential BC markers includes extracellular matrix metalloproteinases that involve metalloproteinase 3 (MMP-3) and metalloproteinase 7 (MMP-7).

The aim of the present study was to examine the diagnostic utility of MMP-3 and MMP-7 as modern markers of early stages of BC, compared to women with benign lesions and healthy female volunteers, alone and in correlation with an accepted marker—CA 15-3.

In physiological breast tissue, expression of both MMP-3 and MMP-7 was found [37]. Immunochemical staining showed the presence of MMP-3 within glandular cells and myoepithelial cells [38]. Expression of both of these MMPs is also found in various histological types of BC [37,39,40,41]. However, in the case of MMP-3, no differences are found between mRNA levels between physiological breast tissue and cancerous lesions [37,42], while MMP-7 expression is a matter of debate. According to Zhang et al. [41], higher levels of MMP-7 are found in BC than in normal breast tissue, while Köhrmann et al. [37] finds an inverse dependence. In addition, Benson et al. [42] finds no differences between MMP-7 expression in physiological and cancerous tissue, but it should be noted that the study was performed on a small number of patients (n = 39). MMP-3 expression was not dependent on clinicopathological parameters such as lesion size and grade, estrogen receptor expression status and lymph node status [39]. In contrast, MMP-7 expression is found to be higher in more aggressive forms of BC, correlates with tumor size, presence of metastasis to lymph nodes and distant metastasis [20,41]. Patients with high MMP-3 expression had an unfavorable prognosis and the association of MMP-3 mRNA levels with patient prognosis becomes stronger with increasing BC stage [43]. The associations of MMP-7 expression with the prognosis of BC patients are currently an unexplored field; however, we found a report where the expression of MMP-7 was correlated with the 5-year survival rate of patients [20].

To the best of our knowledge, to date, we are the first research team to evaluate the diagnostic utility of MMP-3 and MMP-7 as plasma markers in Luminal A and Luminal B HER2-negative BC. Preliminary studies on MMP-3 and MMP-7 in the diagnosis of BC have been performed by the group of Piskór et al. [10,22] and also in other types of cancer, especially gynecological cancers such as ovarian [25,44] or cervical cancer [29]. Plasma as a study material was selected on the basis of previous studies and due to the methodology of determining the tested compounds. Additionally, the selection of the comparison group (*fibroadenoma*) was due to previous experience, *fibroadenoma* widely recognized in this type of research [10,45,46]. Due to the lack of similar work and the results in this field from other research teams, the discussion will be based mainly on studies previously performed by the other part of our research team. Since all of our patients were in stage I, our results were related to the results obtained in stage I BC patients from the study by Piskór et al. [10,22]. It is worth noting, however, that in our team’s previous work, we observed an increase in plasma MMP-3 [10] and MMP-7 [22] concentrations in stage III–IV breast cancer patients when compared to patients who were found to be at stage I.

According to our current results, there are no differences in MMP-3 levels between the groups studied. Similar results were previously obtained by Piskór et al. [10], studying patients in stage I of BC (without a distinct histological subtype). These data indicate the validity of the study we performed. Interestingly, in the same study conducted by Piskór et al. [10], the concentration of MMP-10 which, like MMP-3, belongs to the group of stromelysins, was significantly higher in women in stage I of BC compared to the control and comparison group. The same study also reported an increase in level of MMP-3 in the sera of patients with increasing stage of BC (but still not significantly different from healthy control), but since all of our patients were in stage I of BC, we were unable to determine whether a similar relationship could be observed for patients with Luminal A and B HER2-negative subtypes of BC. Discordant results were obtained by the team of Balkhi et al. [47], who demonstrated on an Iranian group of patients higher serum levels of MMP-3 in a group of breast cancer patients with respect to a group of healthy women. In this study, additionally, elevated serum MMP-3 concentrations were directly associated with the presence of a homozygous genotype for the MMP-3 gene. Other teams have also noted associations of various polymorphic forms of the MMP-3 gene with lymph node involvement in a population of South Indian women [48], or higher staging and ER/PR-negative BC type in a population of Native American women [49]; however, interpreting the impact of polymorphism is extremely difficult, as the gene polymorphism is greatly influenced by the ethnicity of the patients [11]. Conflicting reports on MMP-3 levels also relate to specific subtypes of BC. A study involving a group of 800 patients reported that serum MMP-3 levels did not differ between subtypes of BC [50], which is in agreement with our results, where we also found no differences between Luminal A and Luminal B HER2-negative BC patients. This is also supported by findings on MMP-3 expression in BC tissues, where the lack of correlation was noted between not only receptor status, but also histological type, lymph node involvement or menopausal status of patients [38,51]. Nonetheless, a study by the team of Olivares-Urbano et al. [52] on 20 patients under observation before and during radiotherapy treatment showed that MMP-3 reached higher levels in patients with higher grade of BC and among ER/PR-negative BC patients. At the same time, it is noteworthy that MMP-3 levels did not correlate with menopausal status, lymph node involvement or Ki67 index in this study, which is in agreement with other reports on MMP-3 expression in BC tissues.

Data on MMP-7 expression according to BC subtype are limited. According to most studies, the highest MMP-7 expression is found in triple negative BC compared to other BC subtypes [53,54]. In the case of basal-like BC subtype, data are conflicting. As reported by Kim et al. [40], McGowan et al. [39] and Cao et al. [20], BC basal-like subtype has the highest higher expression compared to Luminal A (*p* = 0.007) and HER2-overexpressing subtype (*p* = 0.004). On the other hand, however, González et al. [19] find no difference in mRNA levels in BC basal-like and Luminal A subtype. It is unfortunate that there are currently no data on MMP-7 expression in BC of the Luminal B subtype. The only report indicating serum MMP-7 levels changes in relation to molecular subtype showed that elevated levels were associated with breast cancer subtypes with worse prognosis, that is, receptor-negative and HER2 receptor-positive subtypes. However, the researchers did not perform a detailed analysis of the molecular subtypes of breast cancer [55]. In addition, this study did not show significant differences between serum levels of MMP-7 in breast cancer patients and healthy women. Contradictory results were obtained by the team of Aroner et al. [50], who found no significant differences between serum MMP-7 levels and the molecular subtypes of BC, which is in agreement with our results, with no significant differences in levels being observed between the Luminal A and Luminal B HER2-negative BC groups. Although in the case of MMP-7, we showed higher concentrations of this enzyme in patients with BC of Luminal A subtype, Luminal B HER2-negative and in the BC total group compared to healthy women, which is also in agreement with the results provided by Piskór et al. [22], who find higher levels of MMP-7 in patients in stage I of BC compared to healthy women and those with benign lesions. Additionally, in this study, higher concentrations of MMP-7 were found in stage III and IV compared to stage I [22].

According to the results provided by our team, higher concentrations of the comparative marker CA 15-3 are found in women with BC compared to the control and comparison group, which is in agreement with the results obtained by Motyka et al. [45] and Piskór et al. [10]. This indicates that the methodology of our determinations of this CA 15-3 was correct.

We are currently one of the first teams to have evaluated MMP-3 and MMP-7 concentrations after surgery in women with BC. We collected the material 4–6 weeks after surgery due to the occurrence of post-operative cytokine storm, which can falsify the results in MMPs concentrations. This period was selected based on literature data [45]. In our study, the post-operative concentrations of only MMP-7 (but not MMP-3) studied decreased. Similar determinations for serum concentrations of MMP-7 were performed by the team of Katunina et al. [55], who showed no significant differences in pre- and post-operative levels of MMP-7. However, it should be noted that the measurement of MMP-7 was performed at a time interval of 5–15 days after surgery, which may cause falsification of the results. In addition, after the post-operative recovery period, in our previous studies we observed a decrease in CA 15-3 levels in BC patients [45,46] that we did not find in the current study, which may be a direct result of differences in the selection of patients for the study group.

The diagnostic utility of MMP-3 and MMP-7 was evaluated by several diagnostic tools—sensitivity (SE) and specificity (SP), positive (PPV) and negative (NPV) predictive value. The best values of the mentioned parameters in the BC total group compared to the CA 15-3 showed MMP-7. This is in agreement with the results of Piskór et al. [22], who also obtained comparable or higher SE, SP, PPV and NPV values for MMP-7 compared to CA 15-3 in the BC stage I patient group. It should be noted, however, that all the diagnostic parameters we obtained, with the exception of SE, were lower than in the work of Piskór et al. [22]. This is most likely due to the smaller size of the study group in the previous study (n = 40). In the case of MMP-3, the obtained values of diagnostic parameters were mostly lower than for CA 15-3, which is in partial agreement with the results provided by Piskór et al. [10] (in the stage I group). It is unfortunate that we are not able to relate the obtained diagnostic parameter values of BC subtypes. However, it should be noted that, considering the histological subtype of BC, MMP-7 again showed better values of diagnostic parameters.

When we performed combined analyses of MMP-3, MMP-7 and CA 15-3, in two-parametric or three-parametric analysis we observed changes in diagnostic parameters. We achieved the best results with two-parametric analysis—MMP-7 + CA 15-5—which indicates the possibility of using future MMP-7 determinations simultaneously with CA 15-3 as a new panel of markers in the biochemical diagnosis of BC. Our results are again confirmed by the work of Piskór et al. [22], where after performing a combined analysis of MMP-7 and CA 15-3, an increase in almost all diagnostic parameters was found (again in stage I).

The ROC curve was used to determine the diagnostic power of the tests. In our study, the highest values of diagnostic power were again observed for MMP-7, both in the Luminal A and Luminal B HER2-negative BC patient groups and in the BC total group (0.6888; 0.7612; 0.7250, respectively). It should be noted that higher AUC values for MMP-7 compared to CA 15-3 in the BC total group were found in other studies [22]. For MMP-3, we found lower AUC values compared to CA 15-3 (Luminal A: 0.5040; Luminal B HER2-negative: 0.5232; BC total group: 0.50960, respectively). This is not consistent with the study of Piskór et al. [10], who obtained higher AUC values for MMP-3 than for CA 15-3. Again, this suggests a higher potential for MMP-7 in the biochemical diagnosis of BC compared to MMP-3 and CA 15-3. It should be noted that, unlike other research teams, we did not always find an increase in AUC values when performing combined analyses of the MMPs tested with CA 15-3 (two-parametric or three-parametric analysis). This may be due to differences in the homogeneity of the study groups [10,22,45,46].

According to our team’s present results, it seems that MMP-7 may be considered in the future as a modern BC marker, including Luminal A and Luminal B HER2-negative subtypes, as a single parameter or in a combined analysis with CA 15-3. It is unfortunate that our study has some limitations. The most important one is that the cancer patients were in stage I cancer according to TNM classification. Our study is a continuation of the experiments of Piskór et al. [10] and Piskór et al. [22], which determined the initial potential of MMP-7 and MMP-3 in all stages of BC (without distinguishable histological subtype). Moreover, in the future, we plan to perform similar experiments with Luminal A and Luminal B HER2-negative patients who are also in higher stages of BC according to TNM classification. Another limitation is that only Luminal A and Luminal B HER2-negative subtypes were included in the study. Among other things, we did not include triple-negative BC in the study, which has an extremely unfavorable prognosis. However, triple-negative breast cancer, more often affects young women [56], and our cancer patients were postmenopausal. Nonetheless, we hope that our study will prove useful in the future in the biochemical diagnosis of not only BC of the Luminal A and Luminal B HER2-negative subtypes, but also other BC subtypes.

## Figures and Tables

**Table 1:** Group size and age characteristic of examined preoperative groups: breast cancer total group (BC-total) with distinguished breast cancer Luminal A (Lum A) and Luminal B HER2-negative (Lum B) subgroups of patients and control groups: benign breast lesions (*fibroadenoma*) and healthy women.

		Group Size	Age	
			Median	Range
Breast cancer groups	BC—total	100	59	45–85
	Lum A	50	56	46–65
	Lum B	50	59	48–85
Control groups	*Fibroadenoma*	50	58	45–85
	Healthy women	50	50	44–64

**Table 2:** Plasma concentrations (median and minimum-maximum range) of studied parameters: MMP-3, MMP-7 and comparative marker CA 15-3 in BC patients, *fibroadenoma* subjects and healthy women.

	MMP-3 [ng/mL]	MMP-7 [mg/mL]	CA 15-3 [IU/mL]
Breast Cancer Groups			
**BC—total**	6.61 1.7–18.9	1.49 0.41–11.05	20.16 4.4–151.2
**Lum A**	6.83 1.7–18.9	1.35 0.41–11.05	19.98 6.2–32.3
**Lum B**	6.38 2.35–18.9	1.78 0.55–7.40	20.2 4.4–151.2
Control Groups			
***Fibroadenoma***	5.56 1.98–16.01	1.62 0.25–7.16	16.85 7.5–48.1
**Healthy women**	6.53 1.81–37.33	0.84 0.25–5.23	15.95 6.7–30.2

**Table 3:** Plasma levels (median and minimum–maximum range) prior to and post surgical treatment of MMP-3, MMP-7 and comparative marker CA 15-3 for matched pairs of BC and *fibroadenoma* patients.

Surgical Treatment	MMP-3 [ng/mL]		MMP-7 [ng/mL]		CA 15-3 [IU/mL]	
	Before	After	Before	After	Before	After
Breast cancer group						
**BC—total**	4.34 1.7–15.29	4.32 1.69–15.23	1.41 0.41–11.05	1.01 0.03–9.60	20.22 4.4–151.2	16.9 7.1–31.5
**Lum A**	4.51 1.7–12.16	4.76 1.69–12.95	1.36 0.41–11.05	0.97 0.234–8.5	20.8 6.2–32.3	17.8 8.3–31.5
**Lum B**	4.30 2.35–15.29	3.76 2.09–15.23	1.54 0.55–5.51	1.045 0.03–9.60	17.5 4.4–151.2	16.65 7.1–26.7
Control group						
***Fibroadenoma***	5.4 1.98–16.01	4.42 0.74–16.21	1.25 0.254–7.12	1.21 0.21–6.21	17.7 7.5–48.1	14.1 1.07–39.6

**Table 4:** The Spearman’s rank correlation test for MMP-3, MMP-7 and CA 15-3 in BC group of patients (total group and subgroups) and control groups.

				MMP-3	MMP-7
Breast cancer groups	BC -total	**CA 15-3**	r	−0.0028	0.2583
			*p*	0.9779	0.0102
		**MMP-3**	r	-	0.1333
			*p*	-	0.1861
	Lum A	**CA 15-3**	r	−0.2116	0.1757
			*p*	0.1401	0.2222
		**MMP-3**	r	-	−0.1634
			*p*	-	0.2569
	Lum B	**CA 15-3**	r	0.1527	0.3701
			*p*	0.2897	0.0082
		**MMP-3**	r	-	0.4901
			*p*	-	0.0003
Control groups	*Fibroadenoma*	**CA 15-3**	r	0.1435	−0.1225
			*p*	0.3200	0.3968
		**MMP-3**	r	-	0.2216
			*p*	-	0.1220
	Healthy women	**CA 15-3**	r	−0.0572	0.0922
			*p*	0.6934	0.5241
		**MMP-3**	r	-	0.2412
			*p*	-	0.0915

Red color indicates statistically significant correlations of tested parameters.

**Table 5:** Diagnostic criteria of tested parameters in patients with BC-total group and in selected subgroups.

Tested Parameter	Diagnostic Criteria [%]	Breast Cancer Patients		
		Luminal A Subgroup	Luminal B HER2- Negative Subgroup	Total Group
MMP-3	SE	32	34	33
	SP	80	80	80
	PPV	62	63	77
	NPV	54	55	37
MMP-7	SE	58	70	64
	SP	74	74	74
	PPV	69	73	83
	NPV	64	71	51
CA 15-3	SE	52	56	54
	SP	80	80	80
	PPV	72	74	84
	NPV	63	65	47
MMP-3 + CA 15-3	SE	62	74	68
	SP	64	64	64
	PPV	63	69	79
	NPV	63	72	50
MMP-7 + CA 15-3	SE	74	82	78
	SP	60	60	60
	PPV	65	67	80
	NPV	70	77	58
MMP-3 + MMP-7 + CA 15-3	SE	94	86	90
	SP	14	14	14
	PPV	52	50	68
	NPV	70	50	41

**Table 6:** Characteristics of ROC curve for tested parameters in patients with BC—total group and Luminal A and B subgroups.

Tested Parameter	AUC	SE	95% C.I. (AUC)	*p* (AUC = 0.5)
Breast cancer total group				
CA 15-3	0.6443	0.0451	0.5558–0.7327	0.0040
MMP-3	0.5096	0.0500	0.4116–0.6076	0.8482
MMP-7	0.7250	0.0443	0.6382–0.8119	0.0001
MMP-3 + CA 15-3	0.6468	0.0457	0.5570–0.7365	0.2709
MMP-7 + CA 15-3	0.7246	0.0428	0.6406–0.8085	0.0009
MMP-3 + MMP-7 + CA 15-3	0.7380	0.0418	0.6560–0.8200	0.0006
Luminal A subgroup				
CA 15-3	0.6672	0.0546	0.5600–0.7743	0.0040
MMP-3	0.5040	0.0591	0.3881–0.6198	0.9450
MMP-7	0.6888	0.0532	0.5845–0.7931	0.0011
MMP-3 + CA 15-3	0.6776	0.0539	0.5718–0.7833	0.5705
MMP-7 + CA 15-3	0.7052	0.0519	0.6033–0.8070	0.0207
MMP-3 + MMP-7 + CA 15-3	0.7124	0.0516	0.6112–0.8135	0.0513
Luminal B HER2-negative subgroup				
CA 15-3	0.6214	0.0579	0.5078–0.7349	0.0364
MMP-3	0.5232	0.0591	0.4073–0.6391	0.6893
MMP-7	0.7612	0.0476	0.6679–0.8545	0.0001
MMP-3 + CA 15-3	0.6284	0.0559	0.5187–0.7380	0.3090
MMP-7 + CA 15-3	0.7432	0.0498	0.6454–0.8409	0.0006
MMP-3 + MMP-7 + CA 15-3	0.8040	0.0437	0.7182–0.8898	<0.0001

Red color indicates statistically significant differences when comparing the area under the ROC curve (AUC) to the value of AUC = 0.5, i.e., the limit of diagnostic usefulness of the test.

## Data Availability

The data presented in this study are available on request from the corresponding author.
